# Characterisation data of simple sequence repeats of phages closely related to T7M

**DOI:** 10.1016/j.dib.2016.06.035

**Published:** 2016-06-29

**Authors:** Tiao-Yin Lin

**Affiliations:** Department of Biological Science and Technology, Institute of Molecular Medicine and Bioengineering, National Chiao Tung University, Hsin Chu, Taiwan

**Keywords:** SSR, simple sequence repeat, Simple sequence repeats, T7M, Bacteriophage genome, SSR variability classification

## Abstract

Coliphages T7M and T3, *Yersinia* phage ϕYeO3-12, and *Salmonella* phage ϕSG-JL2 share high homology in genomic sequences. Simple sequence repeats (SSRs) are found in their genomes and variations of SSRs among these phages are observed. Analyses on regions of sequences in T7M and T3 genomes that are likely derived from phage recombination, as well as the counterparts in ϕYeO3-12 and ϕSG-JL2, have been discussed by Lin in “Simple sequence repeat variations expedite phage divergence: mechanisms of indels and gene mutations” [1]. These regions are referred to as recombinant regions. The focus here is on SSRs in the whole genome and regions of sequences outside the recombinant regions, referred to as non-recombinant regions. This article provides SSR counts, relative abundance, relative density, and GC contents in the complete genome and non-recombinant regions of these phages. SSR period sizes and motifs in the non-recombinant regions of phage genomes are plotted. Genomic sequence changes between T7M and T3 due to insertions, deletions, and substitutions are also illustrated. SSRs and nearby sequences of T7M in the non-recombinant regions are compared to the sequences of ϕYeO3-12 and ϕSG-JL2 in the corresponding positions. The sequence variations of SSRs due to vertical evolution are classified into four categories and tabulated: (1) insertion/deletion of SSR units, (2) expansion/contraction of SSRs without alteration of genome length, (3) changes of repeat motifs, and (4) generation/loss of repeats.

**Specifications Table**

**Table:** 

Subject area	*Biology*
More specific subject area	*Genome evolution and sequence mutations*
Type of data	*Figure, tables*
How data was acquired	*Analysis of genomic sequences*
Data format	*Analyzed*
Experimental factors	*Genome sequences were retrieved from NCBI for analysis.*
Experimental features	*Software (ClustalW, IMEx) and manual analysis of the sequences, manual characterization and analysis*
Data source location	*National Chiao Tung University, Hsinchu, Taiwan*
Data accessibility	*Data are within this article.*

**Value of the data**

• Revealing different types of sequence changes of SSRs by vertical evolution of genomes.
• Detailed SSR distributions may aid in identifying broader patterns of phage evolution.
• Provides a guideline for classification of SSR variations in genome comparisons.
• Variations of SSRs in phages may be applied to phage typing.
• Assists researchers studying T7M, T3, ϕYeO3-12, and ϕSG-JL2 related phages in making sequence comparisons.

## 1 Data

**Fig. 1:**
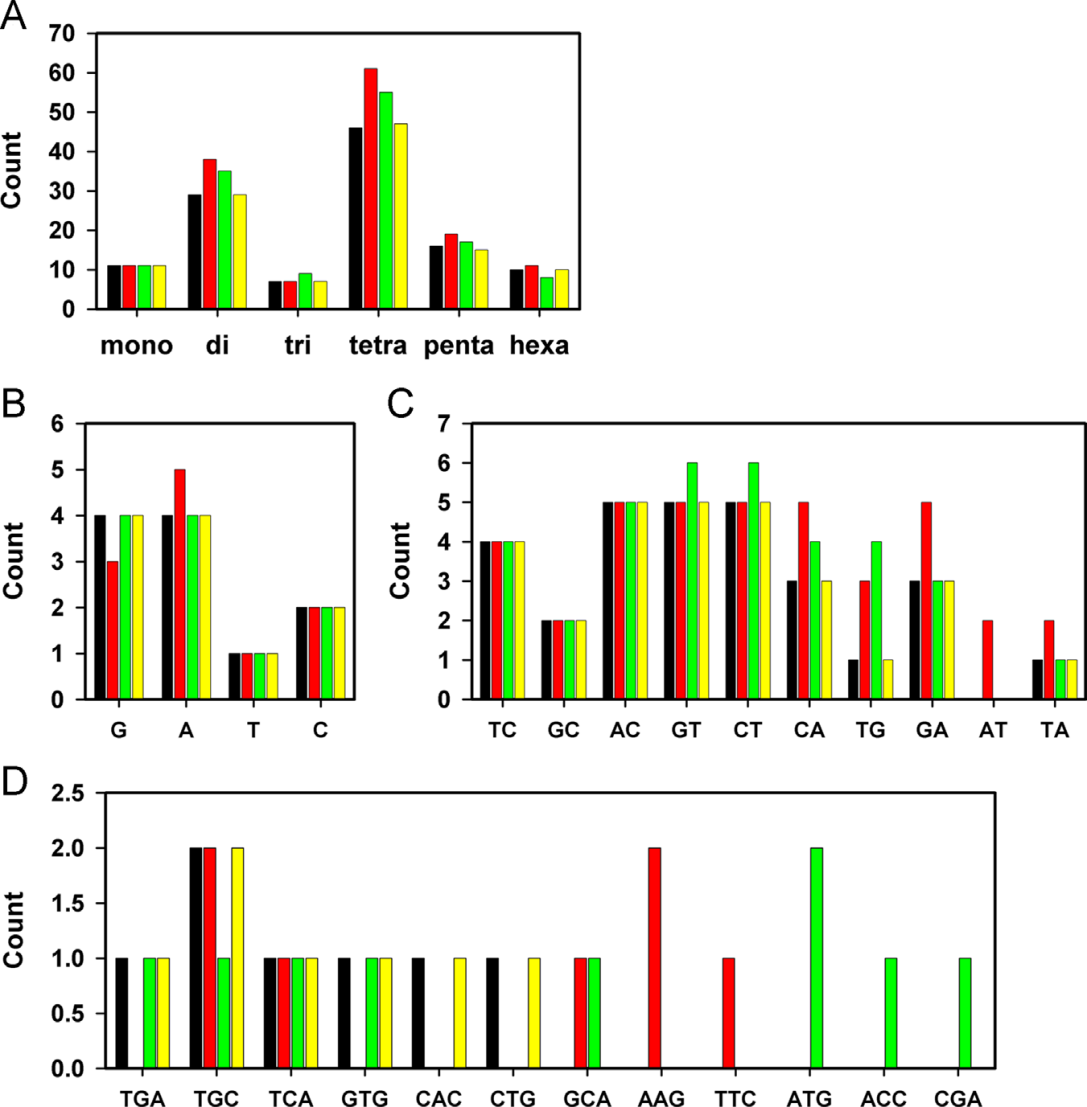
*The distribution of SSR period sizes and motifs in the non-recombinant regions of phage genomes*. SSRs in the non-recombinant regions of T7M and T3 as well as the counterparts in ϕYeO3-12 and ϕSG-JL2 are compared. (A) Counts of mono- to hexanucleotide SSRs. (B) Mononucleotide motifs. (C) Dinucleotide motifs. (D) Trinucleotide motifs. T7M, black; ϕYeO3-12, red; ϕSG-JL2, green; T3, yellow.

Fig. 1 plots the distribution of SSR period sizes and motifs in the non-recombinant regions of the genomes of phages T7M, T3, ϕYeO3-12, and ϕSG-JL2. Table 1 illustrates differences in genomic sequences between T7M and T3. Table 2, Table 3 provide SSR counts, relative abundance, relative density, and GC contents in the complete genomes and non-recombinant regions for T7M, T3, ϕYeO3-12, and ϕSG-JL2. The four classes of SSR variations, (1) insertion/deletion of SSR units, (2) expansion/contraction of SSRs without alteration of genome length, (3) changes of repeat motifs, and (4) generation/loss of repeats, in T7M non-recombinant regions relative to counterpart regions of ϕYeO3-12 and ϕSG-JL2 are tabulated in Table 4, Table 5, Table 6, Table 7, Table 8, Table 9.

## 2 Experimental design, materials and methods

### 2.1 Genome sequences and recombinant regions

The genome sequence of T7M is in NCBI under the accession number GenBank: JX421753 [1]. Genome sequences of ϕYeO3-12, ϕSG-JL2, and T3 are acquired from GenBank accession numbers GenBank: AJ251805 [2], GenBank: NC_010807 [3], and GenBank: AJ318471 [4], respectively. Sequences were aligned by ClustalW [5], and differences between phages are compared. The T7M sequence nt 13245-16687 and 26695-35789 align to T3 nt 13243-16685 and 26700-35794, respectively, and likely arise from a recombination between a ϕYeO3-12-like phage and a T7-like phage, as suggested for T3 [4]. These regions and the counterparts in ϕYeO3-12 and ϕSG-JL2 are referred to as recombinant regions, and the rest of the genomes are referred to as non-recombinant regions [1].

### 2.2 Simple sequence repeats

Simple sequence repeats were searched in phage genomes or non-recombinant regions by IMEx [6]. Unless otherwise specified, the minimum repeat units for mono- to hexanucleotide were 5, 3, 3, 2, 2, 2. Repeats sequences were not standardized.

## Figures and Tables

**Table 1:** Difference in genomic sequences between T7M and T3.

**T7M nt**	**T7M**→**T3 change**	**Location**	**Amino acid change**^a^
26-27	Insertion of C	Terminal repeat	
9606-9607	Deletion of CG	Gene *3*	GVRKVG→CTQGR
9627	Deletion of G	Gene *3*	
9971	Deletion of G	Gene *3*	WL→GV
9975-9976	Insertion of G	Gene *3*	
22153	C→T	Gene *10B*	T→I
22171	C→T	Gene *10B*	T→I
23105	G→A	Gene *12*	A→T
23156	C→A	Gene *12*	L→I
24245	A→G	Gene *12*	N→D
24659	G→A	Gene *12*	G→R
25496-25497	Insertion of AGGGGGG	Between ϕ*13* and gene *13*	
37998-37999	Insertion of C	Terminal repeat	

a Change from T7M to T3 is shown by single letter codes of amino acids.

**Table 2:** SSR counts, relative abundance, and relative density in the complete genome and non-recombinant regions.

	**Size bp**	**SSR count**	**RA**^a^**kb**^**−1**^	**RD**^b^**bp/kb**	**Size bp**	**SSR count**	**RA**^a^**kb**^**−1**^	**RD**^b^**bp/kb**
	**Complete genome**				**Non-recombinant regions**^c^			
T7M	38202	192	5.0	39.7	25664	119	4.6	37.4
ϕYeO3-12	39600	207	5.2	40.8	26813	147	5.5	43.5
ϕSG-JL2	38815	195	5.0	39.3	26335	135	5.1	40.3
T3	38208	192	5.0	39.9	25670	119	4.6	37.6

a Relative abundance: number of SSRs present in per kb of sequence.

b Relative density: the total length (bp) contributed by SSRs per kb of sequence.

c Excluding the two recombination regions in T7M and T3, and the counterpart regions in ϕYeO3-12 and ϕSG-JL2.

**Table 3:** Nucleotide compositions and GC contents of genomic sequences and SSRs in the complete genome versus non-recombinant regions^a^ of phages.

	**T7M**		**ϕYeO3-12**			**ϕSG-JL2**		**T3**
**Complete genome**								

	**% in complete genomic sequence**							
A	26.4		26.2			26.0		26.4
T	23.7		23.2			23.2		23.7
G	26.5		27.0			27.0		26.5
C	23.4		23.6			23.8		23.4
GC	49.9		50.6			50.9		49.9
	**% in SSRs**							
A	23.5 (-2.9)		25.2 (-1.0)			22.6 (-3.4)		23.4 (-3.0)
T	24.6 (1.0)		22.1 (-1.1)			23.8 (0.6)		24.5 (0.9)
G	26.0 (-0.5)		27.0 (0.0)			27.1 (0.1)		26.2 (-0.3)
C	25.8 (2.4)		25.7 (2.1)			26.5 (2.7)		25.9 (2.5)
GC	51.8 (1.9)		52.7 (2.2)			53.6 (2.8)		52.0 (2.1)

**Non-recombinant regions**^a^								

	**% in non-recombinant regions of genome**							
A	26.1		26.2			26.2		26.1
T	23.5		23.3			23.2		23.5
G	26.6		26.6			26.8		26.6
C	23.8		23.9			23.9		23.8
GC	50.4		50.5			50.6		50.4
	**% in SSRs**							
A	22.8 (-3.3)		25.6 (-0.7)			22.0 (-4.2)		22.7 (-3.4)
T	24.6 (1.1)		22.0 (-1.3)			23.0 (-0.2)		24.5 (1.0)
G	25.7 (-1.0)		25.5 (-1.1)			27.7 (1.0)		25.9 (-0.7)
C	26.9 (3.1)		26.9 (3.1)			27.3 (3.5)		26.9 (3.2)
GC	52.6 (2.1)		52.4 (2.0)			55.0 (4.4)		52.9 (2.4)

Only the sequences of sense strands are considered. The number in parenthesis indicates the percent change compared to the complete genomes or the non-recombinant regions of genomes.

a Excluding the two recombination regions in T7M and T3, and the counterpart regions in ϕYeO3-12 and ϕSG-JL2.

**Table 4:** Indels of SSR repeat units in the non-recombinant regions of T7M and counterparts in ϕYeO3-12 and ϕSG-JL2.

**T7M nt**	**Sequence in phage**	
	**T7M**	**ϕYeO3-12**
26	CCCCCCC	CCCCCC-
25497	GGGGGGGGG	-----GGGG
37998	CCCCCCC	CCCCCC-

	**T7M**	**ϕSG-JL2**

26	CCCCCCC	CCCCCC-
7704	ACACACAC	ACACAC--
25497	GGGGGGGGG	-----GGGG
37998	CCCCCCC	CCCCCC-

**Table 5:** Repeat expansion/contraction without alteration of sequence length in the T7M non-recombinant regions and counterparts of ϕYeO3-12 and ϕSG-JL2.

**T7M nt**	**Sequence in phage**	
	**T7M**	**ϕYeO3-12**
8183	TCACACACGG	TCTCACACTG
10777	GTGTGTG	GCCTGTG
17930	CACCACCACCA	CACCGCCACCA
26004	GCGCGCG	GCGCGAG

	**T7M**	**ϕSG-JL2**

6218	CTGATGATGATGG	CTAATGATGATGG
8183-8192	TCACACACGG	TCGAACACAG
8525-8530	CGGGGG	AAGGGG
11576-11584	GTGGTGGTG	GTGGTGGCG
17930-17940	CACCACCACCA	CACCGCCACCA
26004-26010	GCGCGCG	GCGCGAG

Repeat unit is underlined.

**Table 6:** Repeat motif changes in the non-recombinant regions of T7M compared to counterpart regions of ϕYeO3-12.

**T7M nt**	**T7M**	**ϕYeO3-12**
1930	ACGCAGGCAGCAGG	ACGCAGGACGCAGG
4125	GTATCTATC	GTATATACC
5919	CAACGAAATGAAATC	CAACGAAACGAAATC
6218	CTGATGATGATGG	CTAATAATGATGG
8178	GTCACTCACA	GCTACTCTCA
11627	CTTTCGTCCGTCA	CGTTCGTTCGTCA
12316	GGAGAAGGAGAAGGAGA	GAAGAAGGAGAAGGAGA
12700	AATCAATCAAGCAC	AGTCAATCACTCAC
17742	GACATAACATAG	GTCATAGCATAG
19669	TGCTGCTGCCA	TGCAGCAGCAC
20456	CTGCTGCTGCTG	CGGCTGCGGCTG
21313	CTGGCTGGTCTTGT	CTTGCTGGTCTGGT
24066	ACCCATACCCTTCCTT	ACCCATACCCATCGTT
24935	AAGGGTAGGGT	AAGGGTAGAGT
26592	TCCGGGGGA	TCAAAGGTA

SSRs and surrounding sequences are listed. Repeats in ϕYeO3-12 that have at least 3 copies for a mononucleotide or 2 copies for longer repeat periods, but different motifs from those in T7M, are considered. The repeat units with differing motifs between the two phages are underlined.

**Table 7:** SSR generation in the non-recombinant regions of T7M compared to counterpart regions of ϕYeO3-12.

**T7M nt**	**T7M**	**ϕYeO3-12**
1857	GACCGACC	GGATGAAC
7220	GCTGACTGAA	ACTGAGTGAA
9237	CCAAGACAAGAA	CCAAGATAAGAA
9965	AGTGGCGTGGCT	GGTGGAGTGGCT
10159	GGCTGGCTGG	GGCTGGTTAG
11106	TCTGGTCTGGTGGT^a^	TCTGGTCTGGCGGT
11576	GTGGTGGTG	GTGGAGGCG
19278	AATTGCAATTGC	AACTGCAATTGC
20211	GCAGGCAG	GCAGGCCG
20350	TCAGGTCAGG	TCCGGTCAGG
25654	GCTGTGCTGTC	GCTGTGTTGGC
25892	GTCAATTTCAATT	GTCAATTTCAACT
26016	CAGACAGA	CAGACCGA
36359	CCAACCAAC	TCAACCGAC
37140	GCGTTAGCGTTAG	GCGTTAGCATTGG

The newly generated repeat unit in T7M is underlined. The repeat sequence displays at least 3 iterations of a mononuceotide repeat unit or 2 contiguous iterations of a di- to hexanucleotide repeat unit. Repeat sequences in ϕYeO3-12 that are also present in T7M are not considered.

a The sequence has a newly generated GGT repeat in addition to a motif change CTGGT, and both are underlined in this table.

**Table 8:** Repeat motif changes in the non-recombinant regions of T7M compared to counterpart regions of ϕSG-JL2.

**T7M nt**	**T7M**	**ϕSG-JL2**
4125	GTATCTATC	GTGTCTACC
5088	AGCTGCTGGCTGCTG	AGCTGCTAGCTGCTG
11627	CTTTCGTCCGTCA	CGTTCGTTCGTCA
12316	GGAGAAGGAGAAGGAGA	GAAGAAGGAGAAGGAGA
17593	CGATGACGATGA	CGATGATGACGA
17742	GACATAACATAG	GTCATAGCATAG
19669	TGCTGCTGCCA	TGCAGCAGCAC
20456	CTGCTGCTGCTG	CGGCTGCGGCTG
21313	CTGGCTGGTCTTGT	CTGGCTGGTCTGGT
24066	ACCCATACCCTTCCTT	ACCCATACCCATCCTT
24935	AAGGGTAGGGT	AGGGGTAGAGT
26592	TCCGGGGGA	TCAAAGGTA
37648	TACTTACTGCT	TACTTGCTGCT

SSRs and surrounding sequences are listed. Repeats in ϕSG-JL2 that have at least 3 copies for a mononucleotide or 2 copies for longer repeat periods, but different motifs from those in T7M, are considered. The repeat units with differing motifs between the two phages are underlined.

**Table 9:** SSR generation in the non-recombinant regions of T7M compared to counterpart regions of ϕSG-JL2.

**T7M nt**	**T7M**	**ϕSG-JL2**
1930	ACGCAGGCAGCAG	ACGCAGGCCAAGG
4996	GGCTGGCTATAT	GGCTGGTTATAT
5582	AACCTGAACCTG	AAGCTGAACCTA
5731	ACTTTCTTTA	long^a^
5919	CAACGAAATGAAATC	long^a^
8178	GTCACTCACA	GTCACTCGAA
9237	CCAAGACAAGAA	CCAAGATAAGAA
9965	AGTGGCGTGGCT	GGTGGAGTGGCT
10159	GGCTGGCTGG	GGCTGGTTAG
11106	TCTGGTCTGGTGGT^b^	TCTGGTCTGGCGGT
12700	AATCAATCAAG	AGTCAATCACC
16958	ATCAAGCAAGG	ATTAAGCAAGG
19278	AATTGCAATTGC	AACTGCAATTGC
20211	GCAGGCAG	GCAGGCCG
20350	TCAGGTCAGG	TCCGGTCAGG
25654	GCTGTGCTGTC	GCTGTGTTGGC
25892	GTCAATTTCAATTA	GTCAATTCCAATTA
26016	CAGACAGA	CAGACCGA
26335	CAAGTCAAGTC	CGAGTCAAGTC
36359	CCAACCAAC	TCAACCGAC
37140	GCGTTAGCGTTAG	GCGTTAGCATTGG

The newly generated repeat unit in T7M is underlined. The repeat sequence consists of at least 3 iterations of a mononuceotide or 2 contiguous iterations of a di- to hexanucleotide. Repeat sequences in ϕSG-JL2 that are also present in T7M are not considered.

a The sequence is longer in ϕSG-JL2 and does not align well to that of T7M in this region.

b The sequence has a newly generated GGT repeat in addition to a motif change CTGGT, and both are underlined in this table.
